# Supplementary material for: A regulatory network of Sox and Six transcription factors initiate a cell fate transformation during hearing regeneration in adult zebrafish
Source: Cell Genom. 2022 Aug 22;2(9):100170. doi: 10.1016/j.xgen.2022.100170 (PMC9540346; doi:10.1016/j.xgen.2022.100170)
Supplement: Document S1. Figures S1–S9 and Table S15 [file mmc1.pdf]

Cell Genomics, Volume *2*

Supplemental information

A regulatory network of Sox and Six transcription factors initiate a cell fate transformation during hearing regeneration in adult zebrafish

Erin Jimenez, Claire C. Slevin, Wei Song, Zelin Chen, Stephen C. Frederickson, Derek Gildea, Weiwei Wu, Abdel G. Elkahloun, Ivan Ovcharenko, and Shawn M. Burgess

SUPPLEMENTAL ITEM TITLES AND LEGENDS

Figure S1

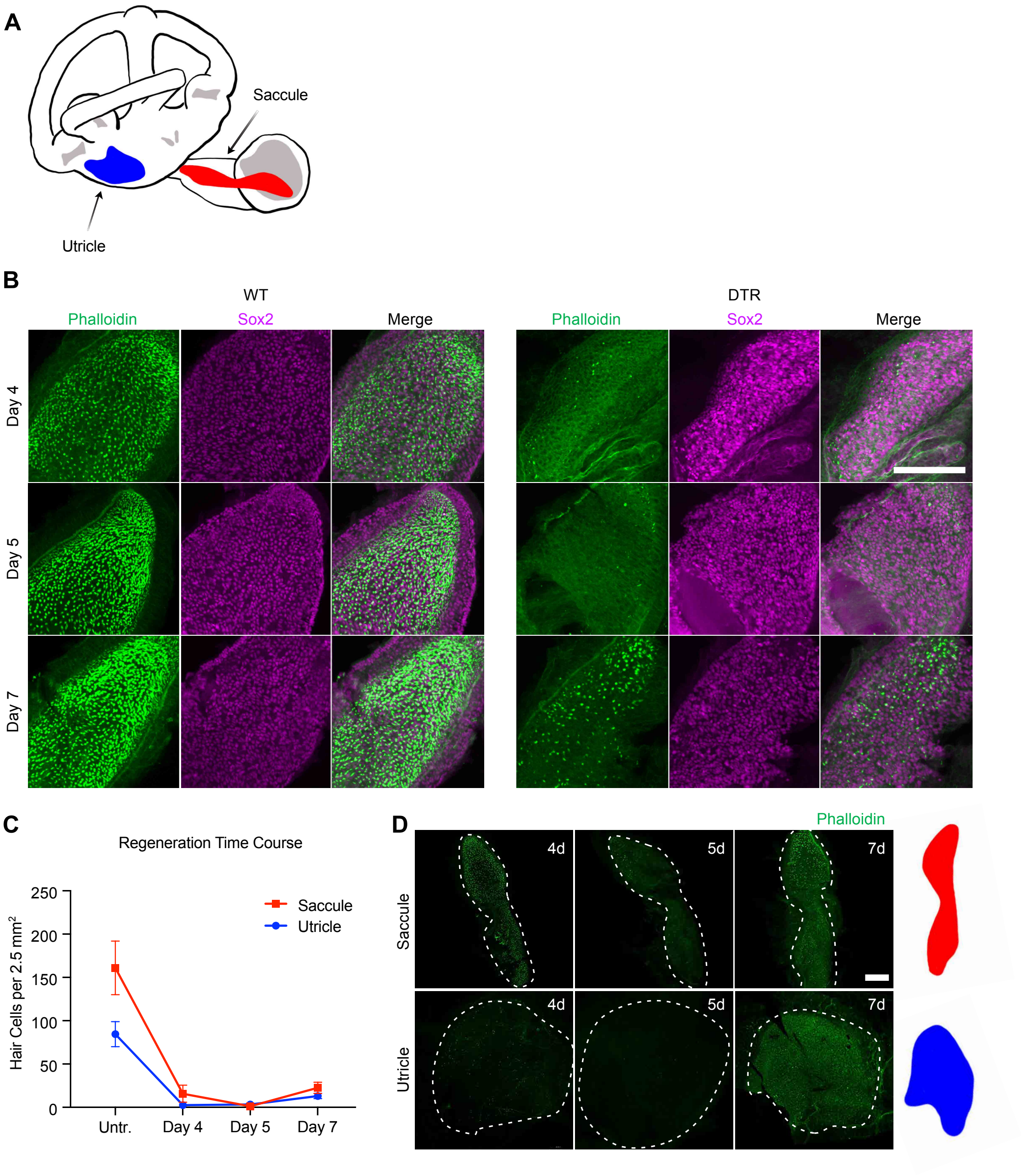

Figure S1. (Related to Figure 1) Vestibular and auditory hair cell regeneration following targeted ablation with diphtheria toxin in zebrafish. (A) Schematic drawing of the adult zebrafish inner ear. The structures used in this study are colored in blue and red. The utricle (blue) is involved in vestibular functions. The saccule (red) is involved primarily in auditory functions. Labelled in gray are macula and crista. (B) Saccule isolated from wild-type (WT) and heterozygous Tg(*myo6b*:hDTR) (DTR) zebrafish following DT injection on days 4, 5, and 7. Phalloidin staining (green) labels hair cell stereocilia and Sox2 immunostaining (magenta) labels supporting cells. Scale bar 100 µm. (C) Quantification of phalloidin positive hair cell numbers in the saccules and utricles of untreated Tg(*myo6b*:hDTR) zebrafish and DT injected Tg(*myo6b*:hDTR) zebrafish on days 4, 5, and 7. ANOVA comparison of the data obtained on days 4, 5, and 7 with the data obtained from untreated inner ears (saccule or utricle) was performed. p-value was determined by ANOVA and Sidak multiple comparison test, p-value < 0.0001. Error bars show SD. *n* = 6-8 in each group. *n* is the number of biologically independent samples (saccule or utricle). (D) Saccule (top) and utricle (bottom) isolated from DT injected Tg(*myo6b*:hDTR) zebrafish on days 4, 5, and 7. Scale bar 100 µm. A schematic drawing of the saccule (red) and utricle (blue) is shown.

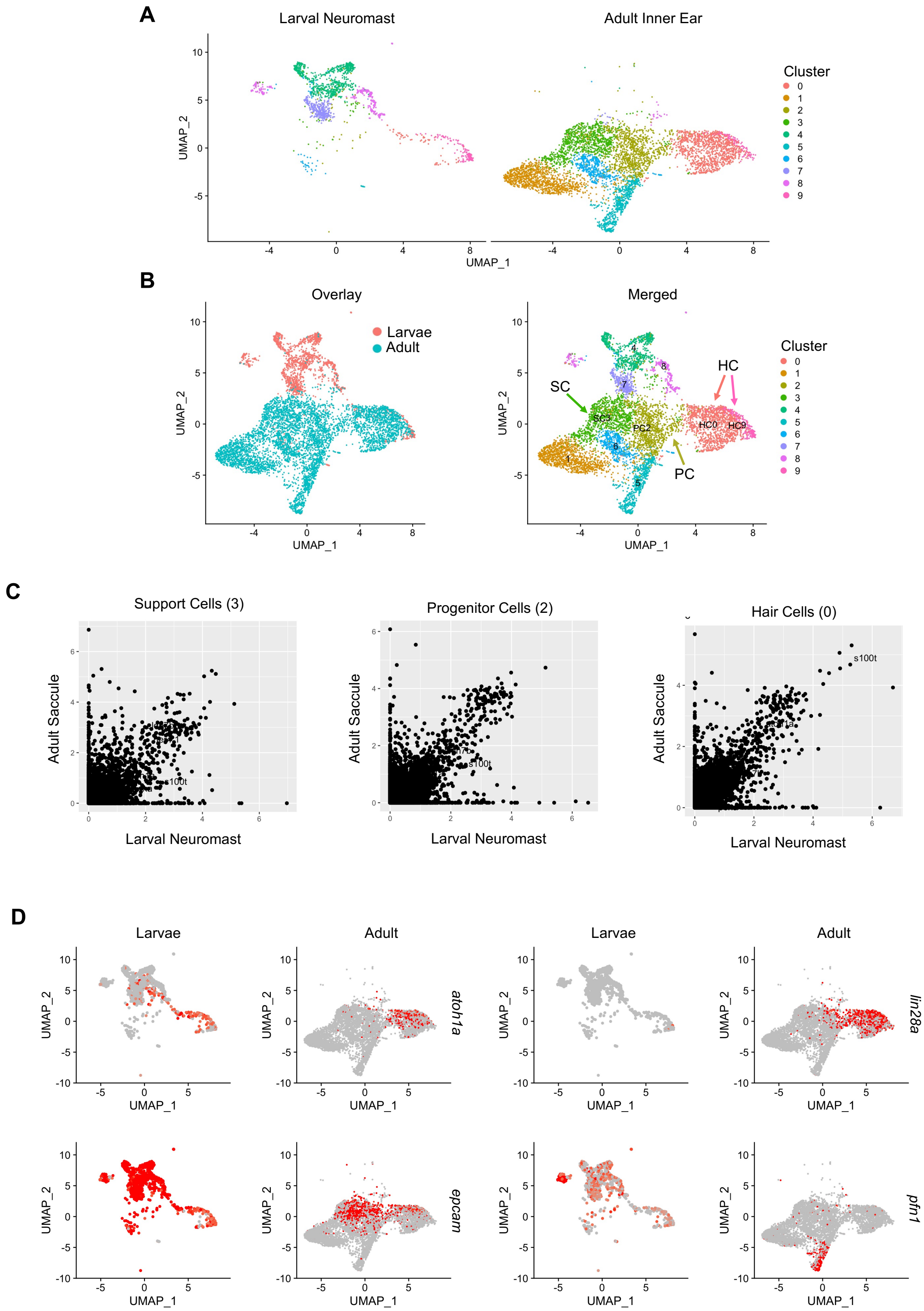

Figure S2. (Related to Figure 1) Adult inner ear cells are transcriptionally distinct from larval lateral line neuromast cells. (A) Unbiased clustering of larval neuromast (left) and adult inner ear tissue (right). (B) Geography of clustered larval neuromast and adult inner ear. (C) Correlation plots comparing cell types. (D) Gene expression profiles of select genes.

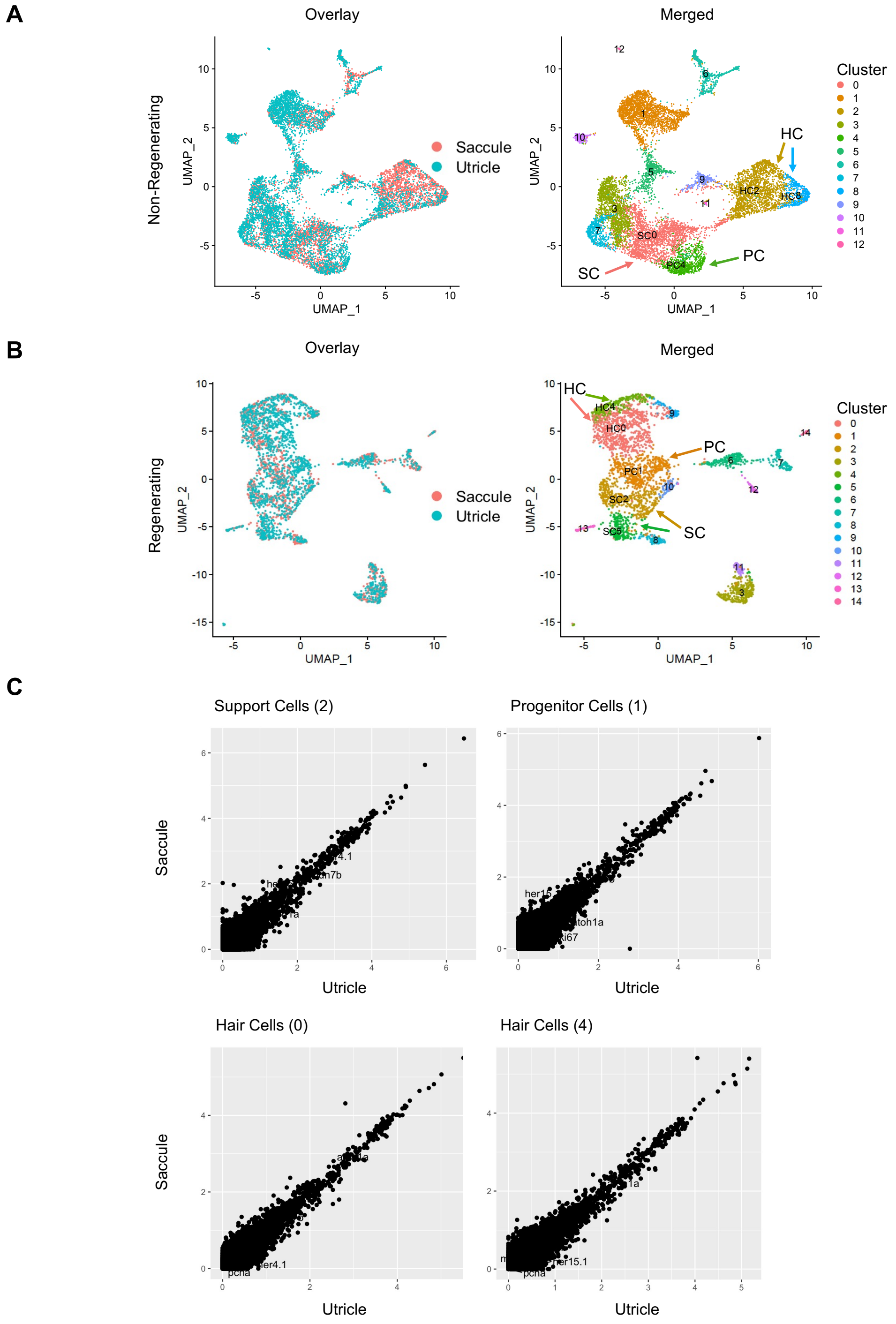

Figure S3. (Related to Figure 1) Organ-level similarities between complementary cell types. (A) Non-regenerating saccule and utricle overlayed (left) and clustered (right). (B) Regenerating saccule and utricle overlayed (left) and clustered (right). (C) Scatter plot analysis of differentially expressed genes in sensory cell types during regeneration. Demonstrates that the organs are highly similar in gene expression.

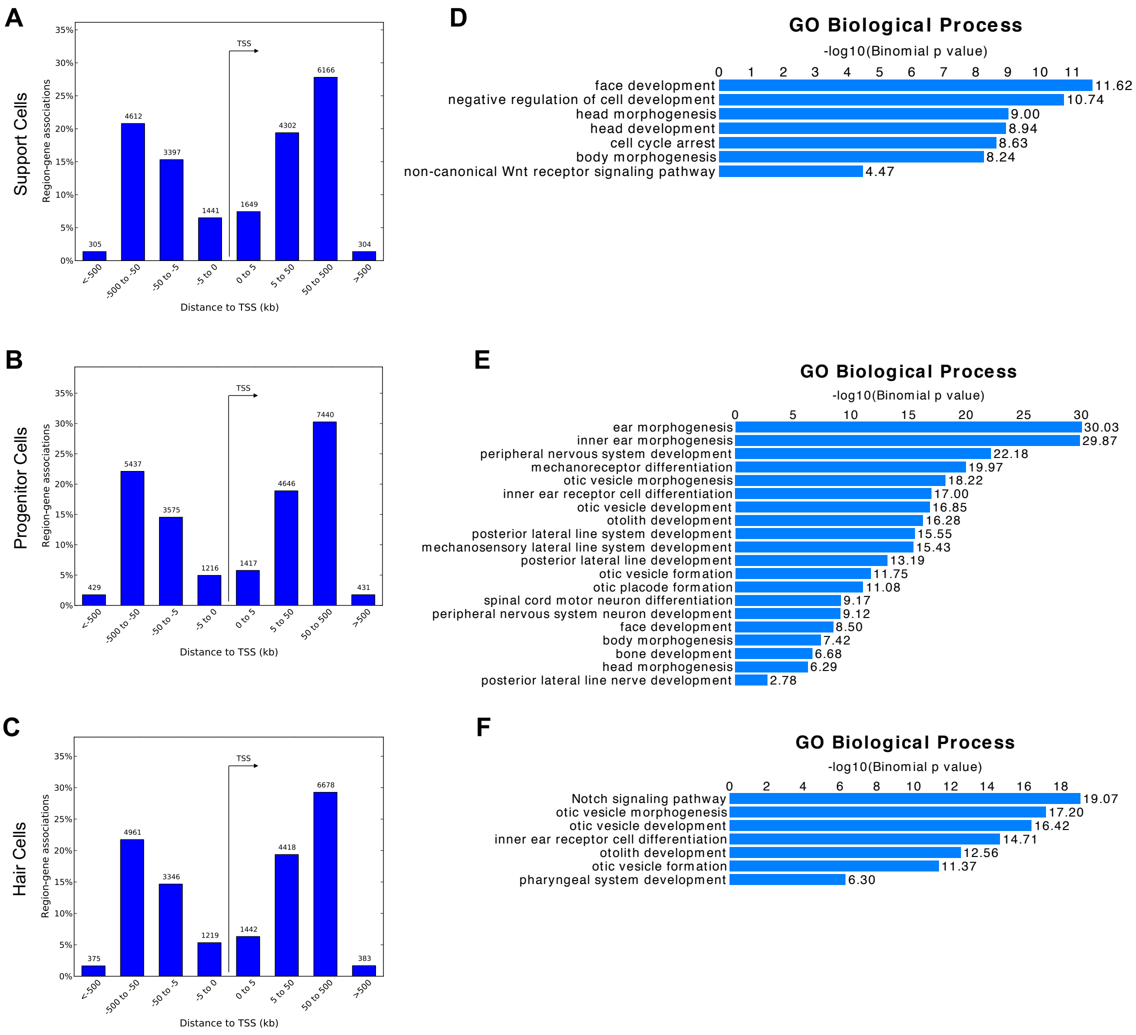

Figure S4. (Related to Figure 5) GREAT analysis on emerging peaks in regenerating inner ear tissues. (A-C) Distance from predicted peak to gene TSS. (D-F) GREAT GO biological processes in predicted cell-specific enhancers.

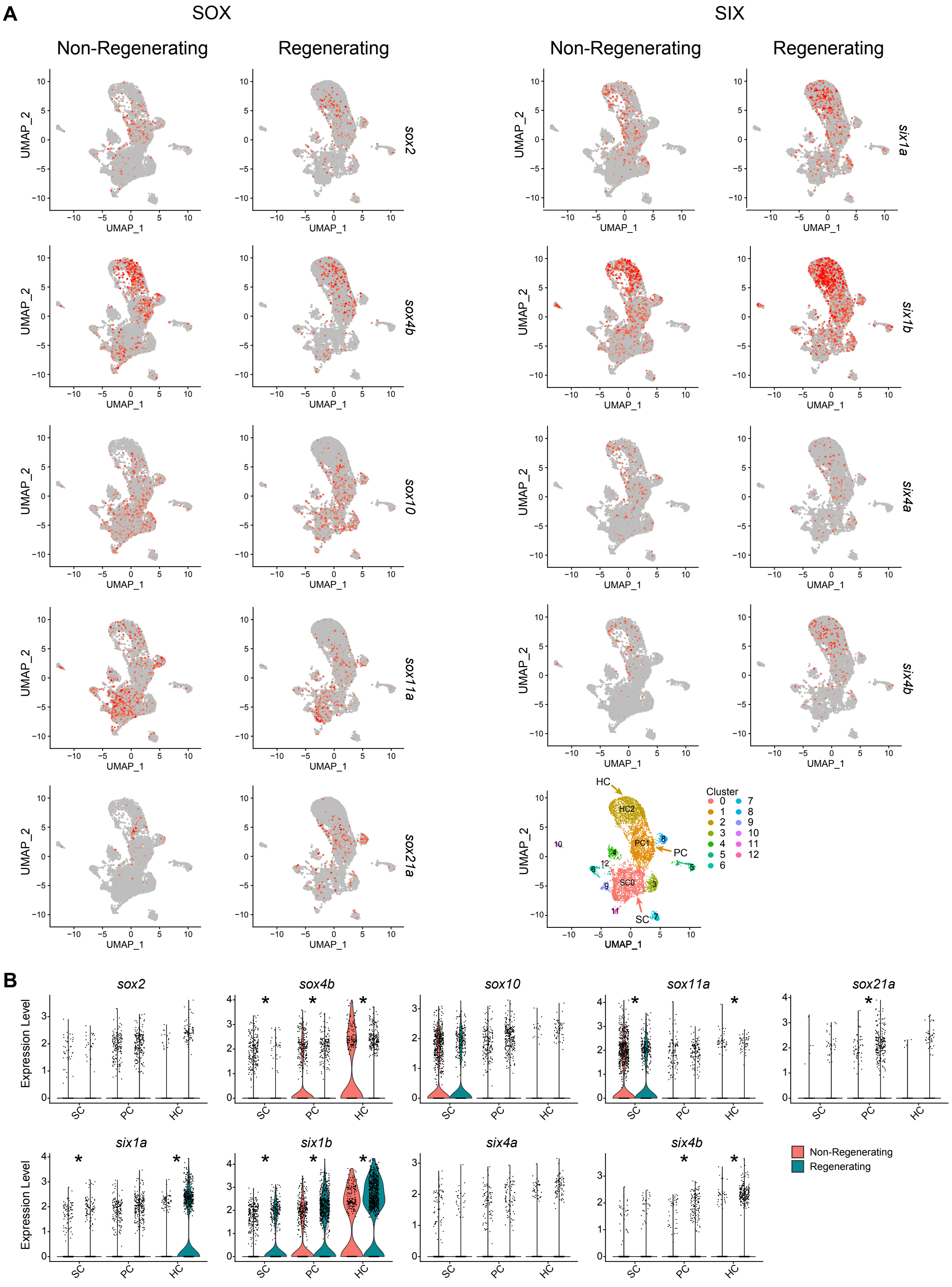
A
SOX
SIX
Non-Regenerating
Regenerating
sox2
sox4b
sox10
sox11a
sox21a
six1a
six1b
six4a
six4b
UMAP_1
UMAP_2
HC
HC2
PC1
PC
SC0
SC
Cluster
B
Expression Level
SC
PC
HC
Non-Regenerating
Regenerating

Figure S5. (Related to Figure 6) Sox and Six TF enrichment corresponds to changes in gene expression during hair cell regeneration. (A) Comparisons between non-regenerating and regenerating inner ear tissues for the identified Sox and Six transcription factors identified as differentially regulated.

UMAP visualization of 8,350 single cells from sensory epithelia (saccule and utricle) from non-regenerating controls include untreated wild-type, untreated Tg(*myo6b*:hDTR) transgenic zebrafish, and wild-type fish injected with DT 4 days post treatment. UMAP visualization of 7,628 single cells from regenerating samples include sensory epithelia (saccule and utricle) from DT injected Tg(*myo6b*:hDTR) transgenic zebrafish on days 4, 5, and 7 post injection. The enrichment of Sox and Six motifs in RREs identified by deep learning correlated to expression of Sox and Six transcription factors during regeneration. UMAP visualization shows annotated cluster identity. Support cells reside in cluster 0 (SC0), progenitor cells reside in cluster 1 (CL1), and hair cells reside in cluster 2 (HC2). (B) Violin plots showing the distribution of gene expression of *sox* and *six* genes across cell types comparing non-regenerating cells (left plot in each cell type) with regenerating cells (right plot). SC = support cells, PC = progenitor cells, and HC = hair cells. Differential (DE) expression testing for cell types between non-regenerating and regenerating datasets was performed using Seurat and the Wilcoxon rank sum test, * = p-value < 0.01; FC ≥ 0.25.

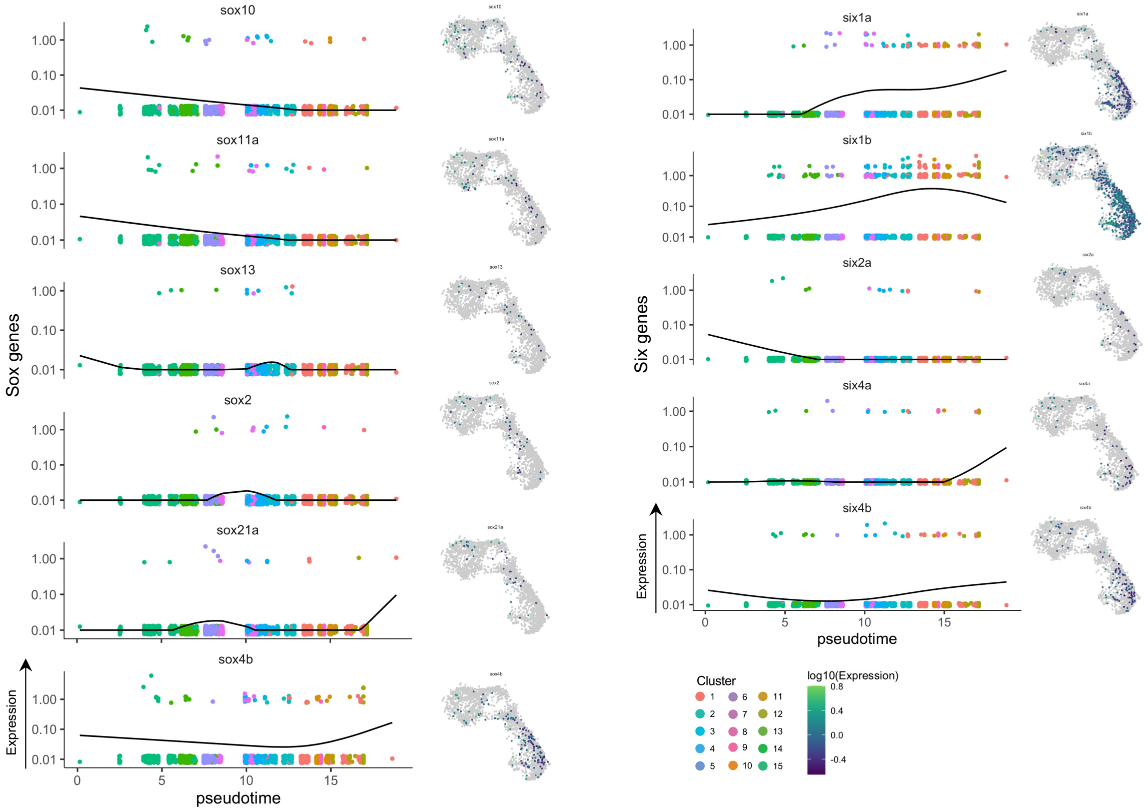

Figure S6. (Related to Figure 6) *sox* and *six* gene expression alterations during hair cell regeneration. Pseudotime ordered single cell expression trajectories for all Sox (left) and Six (right) genes with detectable differential expression.

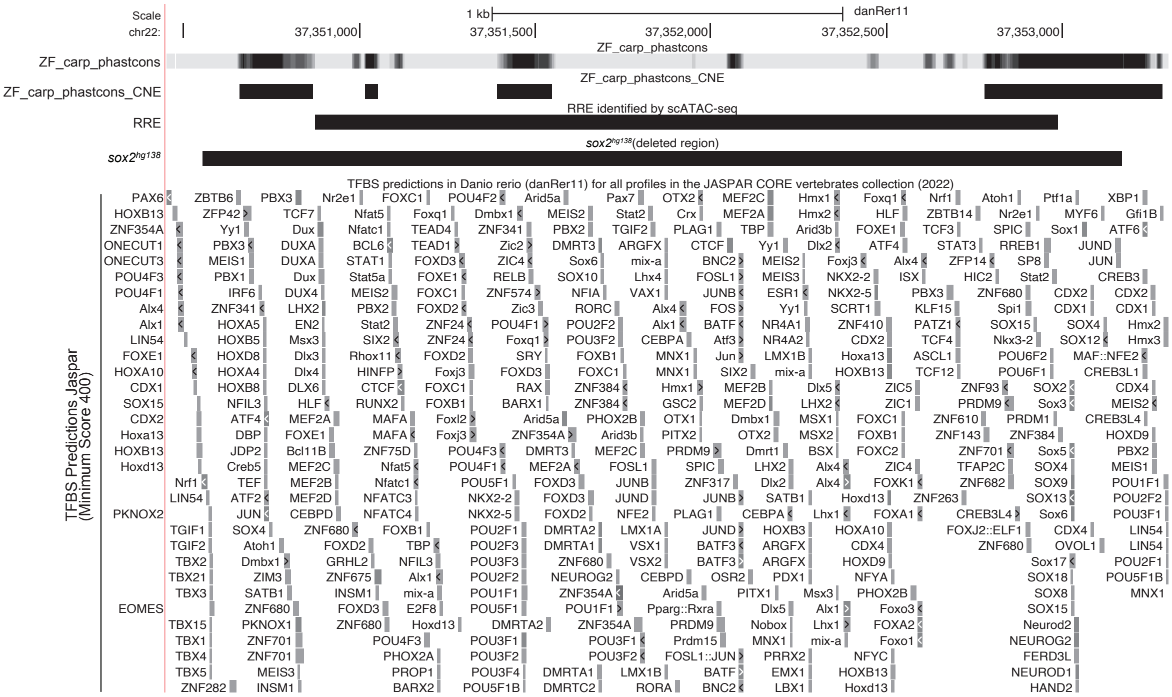

Figure S7. (Related to Figure 7) Predicted TF biding sites for the *sox2*^{hg138} region. UCSC Genome Browser screen shot of the RRE identified using scATAC-seq and the deleted enhancer region in *sox2*^{hg138}. Custom tracks on danRer11 in the UCSC Genome Browser showing the zebrafish/carp/goldfish alignments (ZF_carp_phastcons and ZF_carp_phastcons_CNE). RRE identified by scATAC-seq is located at the genomic coordinates chr22:37,350,875-37,352,990 and the *sox2*^{hg138} deleted region is located at chr22:37,350,554-37,353,171. TFBS predictions in Danio rerio (danRer11) for all profiles in the JASPAR CORE vertebrate collection (2022) are in grey (Minimum score 400).

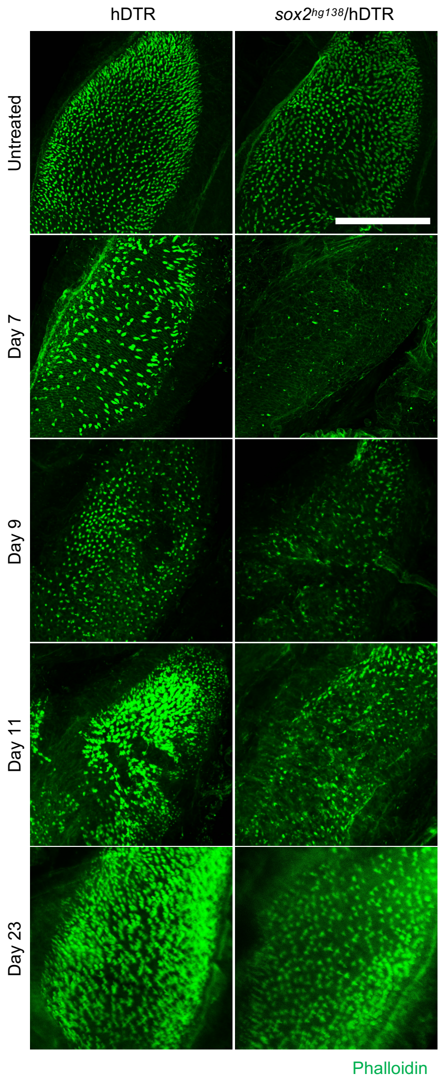

Figure S8. (Related to Figure 7) ***sox2***^{hg138} **enhancer deletion mutants exhibit severely delayed hair cell regeneration in auditory sensory epithelia.** Saccule isolated from heterozygous Tg(*myo6b*:hDTR) and heterozygous *sox2*^{hg138}/hDTR zebrafish following DT injection on days 7, 9, 11, and 23. hDTR = Tg(*myo6b*:hDTR) transgenic zebrafish. Phalloidin staining (green) labels hair cell stereocilia. Brightness and contrast adjusted to 30% and 40%, respectively. Scale bar 100 µm.

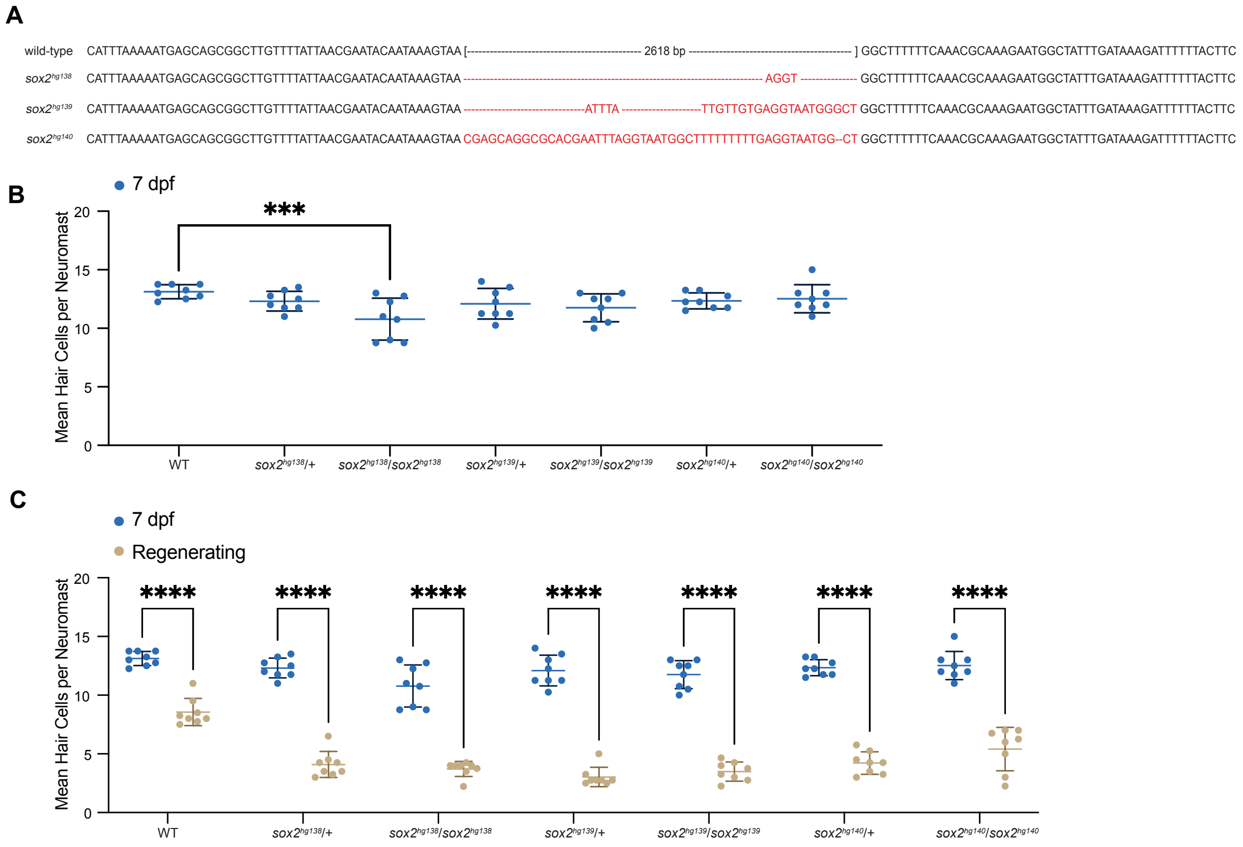

Figure S9. (Related to Figure 7) Independent alleles of *sox2* enhancer deletion mutants exhibit hair cell regeneration defects. (A) Alignment of three independent alleles of *sox2* enhancer deletion mutants: $sox2^{hg138}$, $sox2^{hg139}$, and $sox2^{hg140}$. The maximal targeted region defined by the CRISPR sgRNA guides for deletion is 2618 bp. Any additional sequences in the deletion alleles are marked in red. (B) Mean hair cells per neuromast 7 days post fertilization (dpf) in wild-type (WT), heterozygous and homozygous *sox2* enhancer deletion mutants ($sox2^{hg138}$, $sox2^{hg139}$, $sox2^{hg140}$). The difference in mean hair cells per neuromast in homozygous $sox2^{hg138}$ vs. WT is statistically significant. p-value was determined by ANOVA and Sidak multiple comparison test, ***p-value < 0.001. $n = 8$ in each group. n is the number of biologically independent samples (larvae). (C) Lateral line hair cell regeneration is strongly inhibited 2 days after ablation with $CuSO_4$ in homozygous and heterozygous enhancer deletion mutants. The average number of hair cells and standard deviation are shown in the graph. A two-way ANOVA comparison of the data obtained on untreated larvae with the data obtained from $CuSO_4$ treated larvae was performed. p-value was determined by ANOVA and Sidak multiple comparison test, ****p-value < 0.0001. Error bars show standard deviation. $n = 8$ in each group. n is the number of biologically independent samples (larvae).

Table S15 (Related to Figure 6). Convolutional layers and parameters used in supervised deep learning models.

Structure of CNNs for Deep Learning Model

Convolution1D(64, 9)	activation='relu'	kernel_regularizer=L1L2(l1=1e-4, l2=1e-3)	kernal_constraint=max_norm(1)
	BatchNormalization()	MaxPooling1D(9, 3)	Dropout(0.2)
Convolution1D(32, 4)	activation='relu'	kernel_regularizer=L1L2(l1=1e-4, l2=1e-3)	kernal_constraint=max_norm(1)
	MaxPooling1D(4, 2)	Dropout(0.2)	
Convolution1D(32, 4)	activation='relu'	kernel_regularizer=L1L2(l1=1e-4, l2=1e-3)	
	MaxPooling1D(4, 3)	Dropout(0.2)	
Convolution1D(32, 4)	activation='relu'	kernel_regularizer=L1L2(l1=1e-4, l2=1e-3)	kernal_constraint=max_norm(1)
	MaxPooling1D(4, 2)	Dropout(0.5)	
Flatten()			
Loss='binary_crossentropy', optimizer='ADAM', metrics=['accuracy']			
